# Personalized optimal nutrition lifestyle for self obesity management using metaalgorithms

**DOI:** 10.1038/s41598-022-16260-w

**Published:** 2022-07-20

**Authors:** Shizhao Chen, Yiran Dai, Xiaoman Ma, Huimin Peng, Donghui Wang, Yili Wang

**Affiliations:** College of Mathematics and Physics, Beijing University of Chemical Technology, Beijing, 100029 China

**Keywords:** Obesity, Nutrition, Statistics

## Abstract

Precision medicine applies machine learning methods to estimate the personalized optimal treatment decision based on individual information, such as genetic data and medical history. The main purpose of self obesity management is to develop a personalized optimal life plan that is easy to implement and adhere to, thereby reducing the incidence of obesity and obesity-related diseases. The methodology comprises three components. First, we apply catboost, random forest and lasso covariance test to evaluate the importance of individual features in forecasting body mass index. Second, we apply metaalgorithms to estimate the personalized optimal decision on alcohol, vegetable, high caloric food and daily water intake respectively for each individual. Third, we propose new metaalgorithms named SX and SXwint learners to compute the personalized optimal decision and compare their performances with other prevailing metalearners. We find that people who receive individualized optimal treatment options not only have lower obesity levels than others, but also have lower obesity levels than those who receive ’one-for-all’ treatment options. In conclusion, all metaalgorithms are effective at estimating the personalized optimal decision, where SXwint learner shows the best performance on daily water intake.

## Introduction

Precision medicine improves health care outcomes by applying AI techniques to identify the phenotype closely associated with personalized treatment effects^1^. On each patient, electronic medical records, self-reported lifestyle factors, and genetic information are evaluated to predict heterogeneous treatment outcomes^1^. With advent of the Internet age, individual characteristics are collected using wearable devices, social networks, Internet of Things (IoT), and AI-assistants on diagnoses^1^. Treatment-covariates interaction should be tested and selected to provide a better estimation of heterogeneous treatment effects^2^. Statistical inference methods regarding the reliability of estimated personalized optimal treatment decision are more challenging to derive and less frequently investigated in the literature^3^. Q-learning and A-learning are two primary methods to estimate the personalized optimal dynamic treatment regime, but their performances deteriorate under model misspecification^4^. The main challenge facing precision medicine comes from making treatment plans with multiple stages and multiple treatment options and devising algorithms robust to misspecified models^5^. In general, a personalized optimal medical decision-making system lowers health care costs and brings better treatment effects to everyone^1^.

With the advancement of technology, the data that people obtain are more diverse. It is estimated that IoT worldwide will connect 40 billion devices by 2025 and contribute to more intelligent automation in transportation, manufacturing, and healthcare^6^. IoT utilizes Wireless Sensor Network (WSN) technologies to monitor our living environment continuously and seamlessly^7^. A common operating picture (COP) is created by information sharing through platform interconnection to realize more versatile Internet functions^7^. The Internet of Vehicles (IoV) monitors urban traffic using sensors on roadside equipment and micro-controllers in vehicles to improve traffic safety through cloud-edge computing^8^. Cloud-centric IoT combines WSN, Internet, and distributed computing through the interaction between private and public Clouds, which leads to privacy protection issues^7^. Data management in IoT should meet the requirements of high speed, real-time computing, data continuity, and security^6,9^. The Internet of Medical Things (IoMT) monitors physical symptoms with mobile devices and shares this information with hospitals and disease prevention centers while preserving data privacy^10^. For different forms of IoT, blockchain is a reliable and efficient tool to protect users’ privacy and secure communications between parties^11^. BeCome method applies blockchain to prevent unauthorized data access and balance workload via offloading edge computing devices (ECDs) to facilitate real-time data processing tasks^12^. Locality-Sensitive Hashing (LSH) imputes missing data of continuous, discrete, or Boolean types in medical data without sacrificing privacy^13^. LSROM-EH utilizes blockchain, fog computing, and software-defined networking (SON) for task offloading in wireless body area networks (WBANs) based healthcare ECDs to improve computing efficiency^14^. With IoT and ECDs, a detrended fractal dimension (DFD) feature has been proposed using the fractal dimension (FD) of detrended heart-rate signals from wearable devices, which proved effective in differentiating between regular and insomnia groups^15^.

It is well known that obesity is closely related to the occurrence and development of many diseases, such as metabolic disorders^16^, diabetes^17^, arthritis^18^, cardiovascular diseases (CVD)^19^, and COVID-19^20^. In the US, approximately 67$\%$ of adults diagnosed with arthritis are overweight or obese^18^. Under the widespread lockdown caused by COVID-19, e-learning has become the primary form of education. Although electronic devices have made it more convenient to take classes remotely, e-learning could damage the mental and physical health of students, and obesity might be a problem^21^. Moreover, e-learning could lead to lower scores and more fatigue for deaf and hard of hearing (DHH) students^22^. Increased physical activity and weight-loss counseling can help reduce BMI and treat arthritis^18^. Poor diet and lack of exercise may lead to obesity, which has a significant impact on the progression of type II diabetes and the development of disease complications^17^. In Shenzhen, China, it has been found that overweight and obese male adults had three times more odds of progressing into severe COVID-19 than underweight and normal-weight male adults^20^. It has been discovered that adipokine human Resistin (hResistin) was associated with the secretion of low-grade pro-inflammatory mediators and the development of insulin resistance in obesity-related diseases^23^. The correlation analysis was performed by a one-way ANOVA model with Bonferroni correction, and the Shapiro-Wilk test was used for comparing normally distributed subgroups^23^. A study of 1098 adults found that higher body mass index was associated with ankle systolic-blood-pressures (SBP), and the correlation was obtained by linear regression analysis^19^. Diet-induced obesity (DIO) is related to higher intracranial pressure (ICP) and brain disorders^24^. In the US, obesity is estimated to be responsible for $7\%$ of direct health care spending and $14\%$ of all deaths^17^. Therefore, reducing the BMI of overweight and obese individuals leads to lower average health expenditures and lower risk of severe diseases.

Obesity research often focuses on obesity in childhood and adolescence. Nutrigenetics, epigenomics and metabolomics gather patient information to estimate individualized optimal nutritional decisions^25^. A 1035-person study using hierarchical multiple regression found that maternal obesity and household income significantly affected childhood obesity rates^26^. In a study of 400 overweight or obese women, a higher plant-based dietary index (PDI) leads to better metabolic conditions^27^. Given individual genomics information, nutrigenetics analyze the association between genes and the impact of nutrient intake on the disease status to estimate a personalized optimal diet^28^. In East Asia, people with FTO gene variants had higher BMI when they consumed less protein^16^. On the other hand, the impact of nutrient intake on athlete performance can be affected by environmental conditions, such as social and economic factors, lifestyle patterns, physical activity, and food preferences^28^. The composition of macro-nutrients such as carbohydrates, proteins and lipids can be optimized based on personal genomics and digestive conditions^16^. In an intervention study to lower post-meal blood glucose, a personalized optimal recipe calculated from exercise data and gut microbiome conditions proved effective^29^. Moreover, in a 10-week intervention trial of 82 people, using an individualized optimal nutritional regimen resulted in lower caloric intake and thus reduced individual obesity^30^.

Compared with previous studies, our research has made the following contributions. First, we estimate personalized optimal decisions on dietary lifestyle factors, which are easy to implement and stick to. We consider data on dietary habits including daily water intake and the frequency of alcohol, vegetable and high caloric food consumption. We observe that the calculated individualized optimal treatment options vary from person to person. Second, we build prediction models for body mass index and choose random forest as the base learner for metaalgorithms. We analyze the feature importance of these dietary factors in this predictive study and identify vegetable intake frequency as the most important feature. Third, personalized optimal treatment options we calculate using metaalgorithms result in lower levels of obesity than general ‘one-for-all’ recommendations. People who actually receive treatment options that are exactly equal to individualized optimal treatment options have lower levels of obesity. Fourth, we propose novel metaalgorithms SX and SXwint learners, which outperform other metalearners in the analysis of personalized optimal daily water intake. Compared with T and X learners, SXwint learner has the tendency to show larger distance between personalized optimal individuals and the rest. On the other hand, T and X learners tend to show greater distance between personalized optimal individuals and ones receiving ‘one-for-all’ treatment options.

### Data

To better curb the development of obesity-related epidemics, obesity self-management programs should be easy to implement and adhere to. Genomics and microbiome features are more expensive to measure for large populations. We use an obesity database with 2111 observations and 17 dietary or physical lifestyle features collected in Colombia, Peru and Mexico^31^. In the obesity data, *MTRANS* is the usual means of transportation and consists of five levels: Automobile, Motorbike, Bike, Public Transportation and Walking. *CALC* is a binary indicator of alcohol intake and contains two levels: Yes (positive alcohol intake) and No (zero alcohol intake). *TUE* is the time spent on technological devices. *FAF* is the frequency of physical activity. *SCC* is a binary indicator of food calorie monitoring and comprises two levels: Yes and No. CH_2_O is the amount of daily water intake in liters. *SMOKE* is a binary indicator of smoking and contains two levels: Yes (smoke) and No (never smoke). *CAEC* is the frequency of sub-meals between main meals and involves four levels: No, Sometimes, Frequently and Always. *NCP* is the number of main meals in a day. *FCVC* is the frequency of vegetable intake in meals, where FCVC > 2 means positive vegetable intake in every meal and FCVC $\le$ 2 means zero vegetable intake for some meals. *FAVC* is the frequency of consuming high-calorie foods and contains two levels, where Yes and No mean high-frequency and low-frequency intake of high caloric foods respectively. *FHWO* (family history with overweight) is whether family members have histories of obesity and consists of two levels: Yes and No. Age, gender, height and weight are also recorded.

**Table 1:** Description of characteristics for overweight and obese individuals.

Feature	Feature summary				Drop in variance	*P* value
	Minimum	Maximum	Average	Standard deviation		
Continuous feature						
FCVC	1.00	3.00	2.43	0.51	165.15	0.0000
NCP	1.00	4.00	2.64	0.73	3.40	0.0334
FAF	0.00	3.00	0.93	0.80	3.34	0.0353
CH_2_O	1.00	3.00	2.06	0.60	0.18	0.8328
TUE	0.00	2.00	0.62	0.58	0.15	0.8581
Age	15.00	56.00	25.60	6.48	0.02	0.9784

Categorical feature						
	Category		Count			
FHWO	Yes		1442		17.77	0.0000
	No		102			
FAVC	Yes		1429		12.34	0.0000
	No		115			
SCC	Yes		38		3.48	0.0309
	No		1506			
Gender	Female		720		2.41	0.0897
	Male		824			
CALC	Frequently		51		0.97	0.3793
	Sometimes		1079			
	No		414			
CAEC	Always		17		0.77	0.4629
	Frequently		38			
	Sometimes		1451			
	No		38			
SMOKE	Yes		30		0.75	0.4723
	No		1514			
MTRANS	Walking		18		0.26	0.7674
	Biking		3			
	Public Transportation		1154			
	Automobile		364			
	Motorbike		5			

*Drop in Variance* is the test statistic of covariance test. *P Value* is the p value of covariance test. For continuous features, minimum, maximum, average and standard deviation are displayed. For categorical features, *Count* is the sample size under each category.

BMI (body mass index) is an obesity measure and $\text {BMI} = \text {Weight}/\text {Height}^2$ ($\mathrm{kg}/\mathrm{m}^2$)^31^. In the sample, BMI values range from 12 to 51. Among them, 272 people have BMI between 12 and 18.5, which are underweight, and 287 have BMI between 18.5 and 24.9, which belong to normal-weight category, 580 people have BMI between 25.0 and 29.9 and are overweight, 351 have BMI between 30.0 and 34.9 and are type I obese, and 297 have BMI between 35.0 and 39.9 and are type II obese, 324 people have BMI between 40 and 51 and are classified as type III obesity. People with BMI over 24.9 are overweight or obese, and for them, lowering their BMI is good for their health.

Based on the values of personal characteristics, the estimated personalized optimal eating and living habits are easy for the general public to implement. Before we estimate individualized optimal nutrition lifestyle to reduce BMI, we perform a predictive study for two purposes: (1) comparing the performances of different machine learning methods on predicting BMI, (2) pinpointing the individual features that have significant impacts on BMI. Predictive models with high accuracy can be used as base learners in metaalgorithms to estimate the personalized optimal decision^32^. The significant dietary factors that people can actively change are considered as treatments of interest in pursuit of a personalized optimal nutrition policy. Decision tree methods show higher accuracy in predicting obesity levels than Bayesian and Logistic classification techniques^33^. Among all the features in obesity data, CALC, CH_2_O, FCVC and FAVC measure the intake of alcohol, water, vegetables, and high caloric foods respectively. We use metaalgorithms^32^ to calculate individualized optimal intake regimens for these foods and beverages.

### Predictive model

Catboost^34^ and random forest^35^ are ensemble learning methods for categorical and continuous features. We randomly divide the original data into training and testing set with equal sample sizes. Catboost and random forest models are estimated on the training data and BMI predictions are computed on the testing data. In catboost, we specify the number of iterations to be 200, learning rate as 0.05 and tree depth as 10. The mean absolute error of catboost is 1.36 on the training data, and 2.25 on the testing data. In random forest^36^, we specify the number of tree estimators to be 100, and the minimal number of observations required at each split as 7. The mean absolute error of random forest is 1.13 on the training data and 2.27 on the testing data.

Lasso penalized regression performs model estimation and variable selection simultaneously. Covariance test^37^, one of the mainstream post-selection inference methods, is conditional on the solution path of lasso penalized regression. Each time a new variable is added, model error variance decreases, and the importance of the variable is measured by the magnitude of the decrease^37^. Covariance test is designed for high-dimensional data, but is also suitable for low-dimensional data. Moreover, the obesity data fully meet the assumptions of using covariance test^31^. Lasso penalized regression is to regress BMI on the features in Table 1. P values for variables are derived from the standard exponential distribution Exp(1)^37^. For overweight and obese people, at the 5$\%$ significance level, the following features are significant: FCVC, FHWO, FAVC, SCC, NCP and FAF.

**Figure 1:**
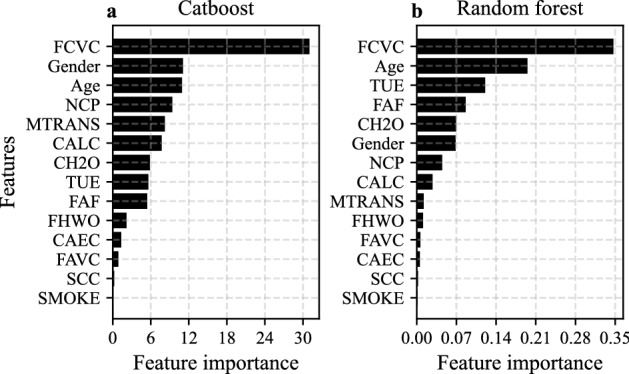
Feature importance for predicting BMI of overweight and obese people using; (**a**) catboost, (**b**) random forest.

According to Fig. 1, for overweight or obese people, both catboost and random forest models regard the following variables to be important: FCVC, TUE, NCP, Age, Gender, FAF, CH_2_O (daily water intake), MTRANS and CALC (alcohol intake frequency), and the following variables to be unimportant: SCC, SMOKE, CAEC, FAVC and FHWO. The variables that covariance test considers important are very different from catboost and random forest models. For example, covariance test considers SCC, FAVC (high caloric food intake frequency) and FHWO to be significant, while catboost and random forest regard them as unimportant. On the other hand, catboost and random forest identify TUE, Age, Gender, CH_2_O, MTRANS and CALC to be important, but covariance test considers them to be insignificant. All three methods identify FCVC (vegetable intake frequency), NCP and FAF (physical activity) to be important. Apparently, among all three methods, significant features found by covariance test are the most intuitive results.

## Methods

Based on the covariance test results in Table 1, we suggest lower frequency of high-calorie food intake, and more physical activity. However, taking other individual covariates into account, the better solution on average is not personalized optimal. For example, quitting alcohol works for some people, but for others, it can be counterproductive. To further reduce BMI, an individualized optimal nutritional regimen should be used instead of better-on-average recommendations. We aim to estimate the personalized optimal decision on alcohol, vegetable, high caloric food and daily water intake. We only consider overweight and obese people with BMI over 24.9^31^. Normal-weight and underweight individuals with BMI less than 24.9 do not require further BMI reductions. Our approach only considers the case of two treatment options. To demonstrate our methods, we view CALC (alcohol intake) to be the treatment *T* of interest. Drinking alcohol at a certain frequency corresponds to CALC $=$ Yes and $T=1$. No alcohol intake corresponds to CALC $=$ No and $T=0$. For the personalized optimal solution of other factors, we replace CALC with these corresponding variables, and then perform the same method.

We use metaalgorithms T, X and S learners to compute personalized optimal scenarios for alcohol intake frequency^32^. Under Neyman-Rubin framework of causal inference^38^, for treatment with binary options, heterogeneous treatment effect is $E\{Y(1)-Y(0)|X=x\}$, where *Y*(1) is the individual BMI outcome for treatment $T=1$, *Y*(0) is the individual BMI outcome for treatment $T=0$, and $X=x$ is the individual covariates that may affect treatment outcomes. If $E\{Y(1)-Y(0)|X=x\}>0$, then $E\{Y(1)|X=x\}>E\{Y(0)|X=x\}$ and treatment $T=0$ is the personalized optimal decision for individuals with covariates $X=x$. If $E\{Y(1)-Y(0)|X=x\}<0$, then $E\{Y(1)|X=x\}<E\{Y(0)|X=x\}$ and treatment $T=1$ is the personalized optimal decision for individuals with covariates $X=x$. If $E\{Y(1)-Y(0)|X=x\}=0$, then treatment $T=0$ or $T=1$ are equally favorable for people with covariates $X=x$. Causal forest estimators of heterogeneous treatment effects have been shown to be point-wise consistent and asymptotically Gaussian distributed^36^. All base learners are specified to be random forest models, since they have decent accuracy in predicting BMI. T, X and S learners all require splitting the obesity data into training and testing data with equal sample sizes. On the training data, the personalized optimal nutrition plan is estimated, and on the testing data, each individual’s personalized optimal nutrition plan can be calculated.

**Figure 2:**
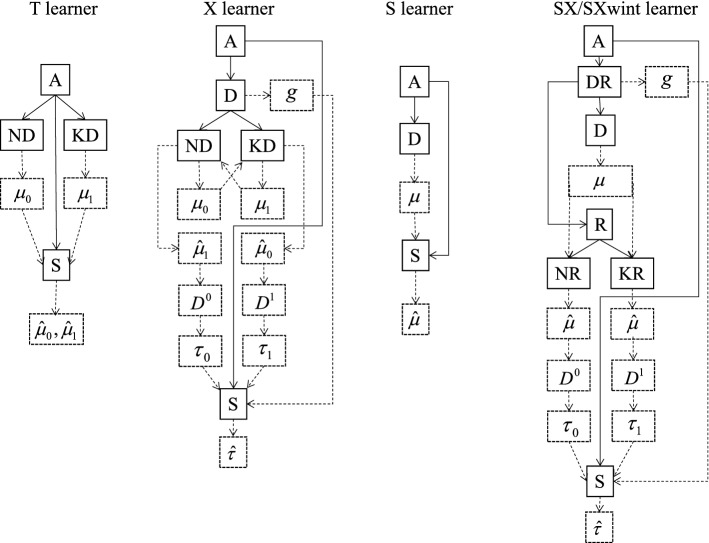
Illustration of metaalgorithms. *D*: Training data. *S*: Testing data. *R*: Re-training data. *A*: The set of all individuals. *DR*: The union of training and re-training data. *N*: The set of all individuals with treatment observation T $=$ 0. *K*: The set of all individuals with treatment observation T $=$ 1. *ND*: Training data from N. *NS*: Testing data from N. *NR*: Re-training data from N. *KD*: Training data from K. *KS*: Testing data from K. *KR*: Re-training data from K. Solid lines represent random splits of datasets. Dotted lines stand for the computational processes of models.

As shown in Fig. 2,* T learner*^32^ has the following steps. (***T1***) Identify the subset of training data where observed treatment $T=0$. Estimate $\mu _0(x)=E(Y(0)|X=x)$ using random forest model ${\hat{\mu }}_0(x)$, where *Y*(0) is the BMI of individuals without any alcohol intake and $X=x$ is the observation of individual covariates. (***T2***) Identify the subset of training data where observed treatment $T=1$. Estimate $\mu _1(x)=E(Y(1)|X=x)$ using another random forest model ${\hat{\mu }}_1(x)$, where *Y*(1) is the BMI of individuals with positive alcohol intake and $X=x$ is the observation of personal characteristics. (***T3***) On the testing data, compute ${\hat{\mu }}_0(x)$ and ${\hat{\mu }}_1(x)$ using covariates *x* of each individual. If ${\hat{\mu }}_1(x)>{\hat{\mu }}_0(x)$, lower BMI is what we want, so $T=0$ and CALC=No is the personalized optimal decision for this individual. If ${\hat{\mu }}_1(x)<{\hat{\mu }}_0(x)$, then $T=1$ and CALC $=$ Yes is the personalized optimal decision for this individual. If ${\hat{\mu }}_1(x)={\hat{\mu }}_0(x)$, then CALC $=$ No and CALC $=$ Yes are equally beneficial for this individual.

As shown in Fig. 2,* X learner*^32^ has the following steps. (***X1***) Perform steps (*T1*) and (*T2*) of T learner on the training data. On the training data with $T=1$, estimate $\mu _1(x)=E(Y(1)|X=x)$ with random forest model ${\hat{\mu }}_1(x)$. On the training data with $T=0$, estimate $\mu _0(x)=E(Y(0)|X=x)$ using another random forest model ${\hat{\mu }}_0(x)$. (***X2***) On the subset of training data where observed treatment $T=0$, we observe *Y*(0) and compute ${\hat{\mu }}_1(x)$, which is an estimate of the potential outcome if treatment $T=1$ were assigned. Compute the difference in outcomes $D^0={\hat{\mu }}_1(x)-Y(0)$. Estimate $\tau _0(x)=E(D^0|X=x)$ using random forest model ${\hat{\tau }}_0(x)$. (***X3***) On the subset of training data where observed treatment $T=1$, we observe *Y*(1) and compute ${\hat{\mu }}_0(x)$, which is an estimate of the potential outcome if treatment $T=0$ were assigned. Compute the difference in outcomes $D^1=Y(1)-{\hat{\mu }}_0(x)$. Estimate $\tau _1(x)=E(D^1|X=x)$ using random forest model ${\hat{\tau }}_1(x)$. (***X4***) On all training data, estimate the propensity score^39^ $g(x)=P(T=0|X=x)$ using random forest model ${\hat{g}}(x)$. (***X5***) On the testing data, compute ${\hat{\tau }}(x)={\hat{g}}(x){\hat{\tau }}_0(x)+\{1-{\hat{g}}(x)\}{\hat{\tau }}_1(x)$. If ${\hat{\tau }}(x)>0$, then $T=0$ and CALC=No is the personalized optimal decision for this individual. If ${\hat{\tau }}(x)<0$, then $T=1$ and CALC=Yes is the personalized optimal decision for this individual. If ${\hat{\tau }}(x)=0$, then CALC=No and CALC=Yes are equally helpful for this individual.

As shown in Fig. 2,* S learner*^32^ has the following steps. (**S1**) On all training data, estimate the joint model $\mu (x,t)=E(Y|X=x,T=t)$ using random forest model ${\hat{\mu }}(x,t)$, where *Y* is the BMI measurement, $X=x$ is the observation of individual covariates and $T=t$ is the observed CALC treatment. Here in $X=x$, we must include all first-order interaction terms between treatment and individual covariates. (**S2**) On the testing data, compute ${\hat{\mu }}(x,0)$ and ${\hat{\mu }}(x,1)$ using covariates *x* of each individual. If ${\hat{\mu }}(x,1)>{\hat{\mu }}(x,0)$, then $T=0$ and CALC=No is the personalized optimal decision for this individual. If ${\hat{\mu }}(x,1)<{\hat{\mu }}(x,0)$, then $T=1$ and CALC=Yes is the personalized optimal decision for this individual. If ${\hat{\mu }}(x,1)={\hat{\mu }}(x,0)$, then CALC=No and CALC=Yes are equally advantageous for this individual.

Based on T, X and S learners, we propose SX and SXwint learners. Both SX and SXwint learners require dividing the obesity data into training, re-training and testing data, which account for 1/4, 1/4 and 1/2 of the obesity data respectively. For datasets with large samples and few features, decomposing the original data into three parts can alleviate over-fit problem. Figure 2 illustrates metaalgorithms T, X, S, SX and SXwint learners. The steps of SX and SXwint learners are the same, except that SXwint learner uses first-order interactions between treatment and covariates but SX learner does not use any. S learner does not split the original data into samples with observed treatment $T=0$ and ones with $T=1$. Instead S learner uses the whole data for joint modeling, which can better distinguish between personalized optimal group and the rest. However, past research results have shown that X learner performs much better than S learner. Our motivation is that we combine the steps of X and S learners to create a new method that inherits the advantages of both. From Fig. 2, we can see that SX and SXwint learners are formed by splicing the steps of S learner and the steps of X learner.

As shown in Fig. 2,* SX learner*^32^ has the following steps. (*SX1*) Execute step (*S1*) of S learner procedures on the training data, where in $X=x$, we do not use any interaction between treatment and covariates. Estimate $\mu (x,t)=E(Y|X=x,T=t)$ using random forest model ${\hat{\mu }}(x,t)$. (*SX2*) Execute step (*X2*) of X learner procedures on the re-training data. On the subset of re-training data where observed treatment $T=0$, we observe *Y*(0) and compute ${\hat{\mu }}(x,1)$. Compute $D^0={\hat{\mu }}(x,1)-Y(0)$. Estimate $\tau _0(x)=E(D^0|X=x)$ using random forest model ${\hat{\tau }}_0(x)$. (*SX3*) Execute step (*X3*) of X learner procedures on the re-training data. On the subset of re-training data where observed treatment $T=1$, we observe *Y*(1) and compute ${\hat{\mu }}(x,0)$. Compute $D^1=Y(1)-{\hat{\mu }}(x,0)$. Estimate $\tau _1(x)=E(D^1|X=x)$ using random forest model ${\hat{\tau }}_1(x)$. (*SX4*) Execute step (*X4*) of X learner procedures. On the union of training and re-training data, estimate $g(x)=P(T=0|X=x)$ using random forest model ${\hat{g}}(x)$. (*SX5*) Execute step (*X5*) of X learner procedures on the testing data.

As shown in Fig. 2,* SXwint learner*^32^ has the following steps. (*SXwint1*) Execute step (*SX1*) of SX learner procedures on the training data. In $X=x$, we use first-order interaction terms between treatment and individual covariates. Estimate $\mu (x,t)=E(Y|X=x,T=t)$ using random forest model ${\hat{\mu }}(x,t)$. (*SXwint2*), (*SXwint3*), (*SXwint4*) and (*SXwint5*) are the same as steps (*SX2*), (*SX3*), (*SX4*) and (*SX5*) of SX learner procedures. In SXwint learner, *x* contains all first-order treatment-covariates interactions, but in SX learner, *x* does not use any interaction. In most cases, SXwint learner performs better than SX learner, as presented in Table 2.

In SX and SXwint learners, we estimate a joint model $\mu (x,t)=E(Y|X=x,T=t)$ rather than $\mu _1(x)=E(Y(1)|X=x)$ and $\mu _0(x)=E(Y(0)|X=x)$ separately. We use training data to estimate the joint model $\mu (x,t)=E(Y|X=x,T=t)$ and re-training data to estimate $\tau _0(x)$ and $\tau _1(x)$ rather than one training set to estimate both. The convergence rates of T, S and X learners have been demonstrated under strict assumptions^32^. It assumes linear heterogeneous treatment effect, base learner estimators have high prediction accuracy under all data distributions $\mathcal {P}$, random errors satisfy $E(\varepsilon |X=x)=0$ and $Var(\varepsilon |X=x)\le \sigma ^2$, features have finite variances, and the covariance matrix is well-conditioned. The obesity data we study^31^ fit these strict assumptions. The convergence rates of SX and SXwint learners are of the same scale as the convergence rate of X learner.

### Remark 1

In S learner, *x* must contain interaction terms between treatment and individual covariates. Under the framework of linear heterogeneous treatment effects, in S learner, without interactions, $\mu (x,t)=x\beta _1+t\beta _2$, treatment effect is $\mu (x,1)-\mu (x,0)=\beta _2$, which is independent of *x*, resulting in the estimation strategy not being personalized. However, in S learner with first-order treatment-covariates interactions, $\mu (x,t)=x\beta _1+t\beta _2+xt\beta _3$, then treatment effect is $\mu (x,1)-\mu (x,0)=\beta _2+x\beta _3$, which contains *x*, so the estimation strategy is indeed personalized. Hence in S learner, *x* must contain treatment-covariates interactions in order for the estimation result to be personalized optimal.

### Remark 2

SX and SXwint learners are equivalent when we specify $g(x)=0$ and ${\hat{g}}(x)=0$. We know that $\tau _0(x)=E(D^0|X=x)=E\left[ {\hat{\mu }}(x,1)-Y(0)|X=x\right] ={\hat{\mu }}(x,1)-\mu _0(x)$ and that $\tau _1(x)=E(D^1|X=x)=E\left[ Y(1)-{\hat{\mu }}(x,0)|X=x\right] =\mu _1(x)-{\hat{\mu }}(x,0)$. In SX and SXwint learners, set $g(x)=0$ and ${\hat{g}}(x)=0$, then treatment effect is $\tau (x)=\tau _1(x)=\mu _1(x)-{\hat{\mu }}(x,0)$. Regardless of whether *x* contains first-order interaction terms or not, we have that $\mu (x,0)=x\beta _1$ and ${\hat{\mu }}(x,0)=x{\hat{\beta }}_1$. Then $\tau (x)=\tau _1(x)=\mu _1(x)-x{\hat{\beta }}_1$. The formulas of treatment effects are equivalent in SX and SXwint learners.

### Remark 3

Regardless of whether *x* contains first-order interaction terms, the policies estimated by SX and SXwint learners are personalized optimal. In SXwint learner, with first-order interactions, $\tau _0(x)={\hat{\mu }}(x,1)-\mu _0(x)=x{\hat{\beta }}_1+{\hat{\beta }}_2+x{\hat{\beta }}_3-\mu _0(x)$ and $\tau _1(x)=\mu _1(x)-{\hat{\mu }}(x,0)=\mu _1(x)-x{\hat{\beta }}_1$. Then treatment effect is $\tau (x)=g(x)\tau _0(x)+\{1-g(x)\}\tau _1(x)=g(x)\{x{\hat{\beta }}_1+{\hat{\beta }}_2+x{\hat{\beta }}_3-\mu _0(x)\}+\{1-g(x)\}\{\mu _1(x)-x{\hat{\beta }}_1\}$, which contains *x*, indicating that the estimated policy is personalized optimal. In SX learner, without any interaction, $\tau _0(x)={\hat{\mu }}(x,1)-\mu _0(x)=x{\hat{\beta }}_1+{\hat{\beta }}_2-\mu _0(x)$ and $\tau _1(x)=\mu _1(x)-{\hat{\mu }}(x,0)=\mu _1(x)-x{\hat{\beta }}_1$. Then treatment effect is $\tau (x)=g(x)\tau _0(x)+\{1-g(x)\}\tau _1(x)=g(x)\{x{\hat{\beta }}_1+{\hat{\beta }}_2-\mu _0(x)\}+\{1-g(x)\}\{\mu _1(x)-x{\hat{\beta }}_1\}$, which also contains *x*, so the estimated policy is personalized optimal.

### Remark 4

If we specify that $g(x)=P(T=0|X=x)$, then treatment effects in SX and SXwint learners are related to $xg(x)=E[X(1-T)|X=x]$, which is the first-order interaction between treatment and individual covariates.

Although our methods perform well, they also suffer from the following limitations. First, metaalgorithms perform well only if the prediction accuracy of the base learner is high. In our research, we find that random forest models have high prediction accuracy and use them as base learners in metaalgorithms. If we fail to find a base learner with sufficiently high prediction accuracy, then metaalgorithms do not perform well. Second, SX and SXwint learners use training, re-training and testing data, which account for 1/4, 1/4 and 1/2 of original data respectively. In ultra-high dimensional data where sample size is 10 and feature dimension is in millions, dividing data into training and re-training data results in lower efficiency of data usage, and higher risk of inaccurate predictive models. Third, SXwint learner uses first-order interactions between treatment and covariates. When there are many categorical features that may take many values, the number of dummy variables and first-order interaction terms can become very large. Then the covariates will have higher dimensionality, making it more difficult to train base learners.

## Results

Obesity data are randomly split into training and testing data with equal sample sizes. After we estimate T, X and S learners on the training data, we predict the personalized optimal treatment decision on the testing data. For SX and SXwint learners, training data used in T, X and S learners are now randomly split into new training data and re-training data with equal sample sizes. We estimate SX and SXwint learners using new training data and re-training data. Afterwards we predict the personalized optimal treatment decision for people on the testing data. In summary, when calculating individualized optimal options for each nutrient, T, X, S, SX and SXwint learners decompose the same testing data into the following parts. The* personalized optimal group* is formed by individuals on the testing data whose treatment observations are exactly identical to the personalized optimal decisions. The* non-optimal group* consists of people on the testing data whose treatment observations are different from the personalized optimal decisions. We compare BMI levels in the personalized optimal group and the non-optimal group to determine whether the estimated personalized optimal decision is effective. Furthermore, the* general optimal group* is composed of individuals on the testing data whose treatment observations are equal to the ’one-for-all’ treatment decision. We compare BMI levels in the personalized optimal group and the general optimal group to determine whether individualized decision-making is superior to general advice.

**Table 2:** Two-sample Kolmogorov–Smirnov (KS) test results concerning alcohol, vegetable, high caloric food and daily water intake.

Learner	KS test 1		KS test 2		No.O	No.NO	No.G
	KS test 1 D	*P* value 1	KS test 2 D	*P* value 2			
**Alcohol intake options: Yes No**							
T	0.5647	< 2.2e−16	0.3892	6.7e−16	272	500	207
X	0.5592	< 2.2e−16	**0.3897**	7.8e−16	269	503	207
S	0.5437	< 2.2e−16	0.3284	2.9e−11	262	510	207
SX	0.5255	< 2.2e−16	0.3082	3.5e−11	352	420	207
SXwint	**0.5843**	< 2.2e−16	0.3355	7.1e−13	331	441	207
**Vegetable intake frequency options: high low**							
T	0.5022	< 2.2e−16	0.1782	3.2e−4	275	497	275
X	0.5594	< 2.2e−16	**0.2216**	4.2e−6	258	514	275
S	0.4165	< 2.2e−16	0.1213	4.2e−2	251	521	275
SX	0.4742	< 2.2e−16	0.1473	6.9e−3	249	523	275
SXwint	**0.5741**	< 2.2e−16	0.1131	4.2e−2	333	439	275
**High caloric food intake options: Yes No**							
T	0.6683	< 2.2e−16	**0.3359**	1.2e−4	170	602	58
X	**0.6722**	< 2.2e−16	0.3267	2.2e−4	162	610	58
S	0.6004	< 2.2e−16	0.2343	2.1e−2	147	625	58
SX	0.5391	< 2.2e−16	0.1675	1.3e−1	305	467	58
SXwint	0.6530	< 2.2e−16	0.2096	3.3e−2	242	530	58
**Daily water intake frequency options: high low**							
T	0.4001	< 2.2e−16	0.1474	7.3e−4	341	431	391
X	0.4282	< 2.2e−16	0.1411	7.6e−4	400	372	391
S	0.3880	< 2.2e−16	0.1342	2.5e−3	352	420	391
SX	0.3150	< 2.2e−16	0.1255	7.2e−3	329	443	391
SXwint	**0.4553**	< 2.2e−16	**0.2057**	1.3e−6	295	477	391

*KS Test 1 D* and *P Value 1* are the test statistic and* p* value of *KS test 1* between the distributions of BMI in personalized optimal and non-optimal groups. *KS Test 2 D* and *P Value 2* are the test statistic and* p* value of *KS test 2* between the distributions of BMI in personalized optimal and general optimal groups. *No. O* is the sample size of personalized optimal group. *No. NO* is the sample size of non-optimal group. *No.G* is the sample size of general optimal group.

The largest KS test distance statistic produced under each type of food or drink is in bold.

**Figure 3:**
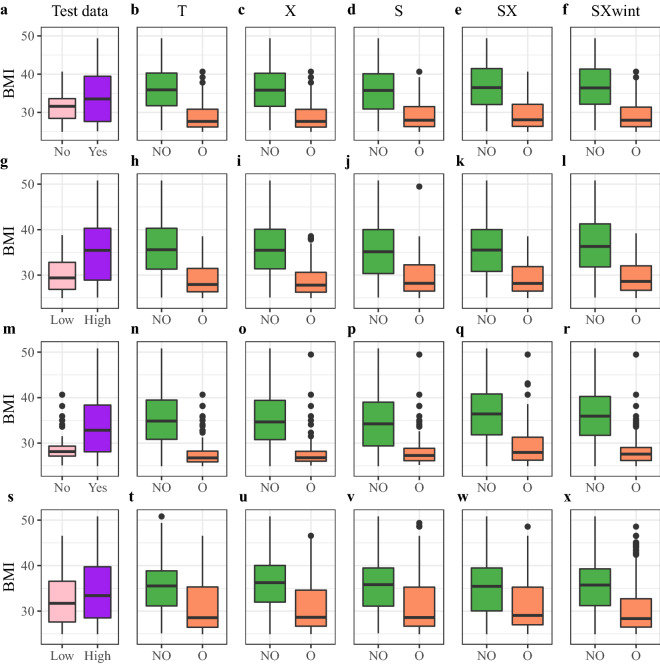
Comparison between BMI distributions in groups; (**a**) Alcohol Yes and No in the testing data, *Yes* = positive alcohol intake, *No* = zero alcohol intake, (**b**) Alcohol T learner NO and O, *NO* = non-optimal group, *O* = personalized optimal group, (**c**) Alcohol X learner NO and O, (**d**) Alcohol S learner NO and O, (**e**) Alcohol SX learner NO and O, (**f**) Alcohol SXwint learner NO and O, (**g**) Vegetable High and Low in the testing data, *High* = FCVC > 2 = positive vegetable intake in every meal, *Low* = FCVC $\le$ 2 = no vegetable intake in some meals, (**h**) Vegetable T learner NO and O, (**i**) Vegetable X learner NO and O, (**j**) Vegetable S learner NO and O, (**k**) Vegetable SX learner NO and O, (**l**) Vegetable SXwint learner NO and O, (**m**) HCF Yes and No in the testing data, *HCF* = high caloric food, *Yes* = high frequency of HCF intake, *No* = low frequency of HCF intake, (**n**) HCF T learner NO and O, (**o**) HCF X learner NO and O, (**p**) HCF S learner NO and O, (**q**) HCF SX learner NO and O, (**r**) HCF SXwint learner NO and O, (**s**) Water High and Low in the testing data, *High* = CH_2_O > 2 = daily water intake greater than 2 liters, *Low* = CH_2_O $\le$ 2 = daily water intake less than or equal to 2 liters, (**t**) Water T learner NO and O, (**u**) Water X learner NO and O, (**v**) Water S learner NO and O, (**w**) Water SX learner NO and O, (**x**) Water SXwint learner NO and O.

In this section, the significance level of hypothesis test results is set to $5\%$ by default. Figure 3 reveals the comparison results between the distributions of BMI in personalized optimal, non-optimal and general optimal groups. Kolmogorov-Smirnov (KS) test is a nonparametric test free of distributional assumption. Two-sample KS test is applied to determine whether two samples follow the same distribution. KS test statistic measures the maximal distance between the empirical BMI distributions of two samples. The distributions of BMI in personalized optimal and non-optimal groups are significantly different according to KS test results in Table 2. In general, for T, X, S, SX and SXwint learners, BMI measurements in personalized optimal groups are significantly lower than BMI levels in non-optimal groups and general optimal groups. Overweight and obese individuals who obey the personalized optimal nutrition plans exhibit much lower BMI measurements than people who follow general ’one-for-all’ recommendations. Hence personalized optimal nutrition regimens are indeed more effective than general suggestions in reducing BMI.

### Learners comparison

For T, X, S, SX and SXwint learners, personalized optimal groups overlap with each other. When calculating the individualized optimal nutrition plan for alcohol intake frequency, within the personalized optimal group of T learner, 83$\%$ people are also personalized optimal in S learner, and 90$\%$ are also personalized optimal in X learner. Among personalized optimal individuals of X learner, 91$\%$ are also personalized optimal in T learner and 83$\%$ are also personalized optimal in S learner. Among personalized optimal individuals of SXwint learner, 93$\%$ are also personalized optimal in SX learner. Among personalized optimal individuals of SX learner, 87$\%$ are also personalized optimal in SXwint learner. When investigating the individualized optimal decisions on alcohol, vegetable, high caloric food and daily water intake, we observe that T and X learners have a greater proportion of overlapping in personalized optimal groups than T and S learners. When analyzing vegetable intake, within the personalized optimal group of SXwint learner, 65$\%$ people are also personalized optimal in SX learner. Among personalized optimal individuals of SX learner, 87$\%$ are also personalized optimal in SXwint learner.

**Figure 4:**
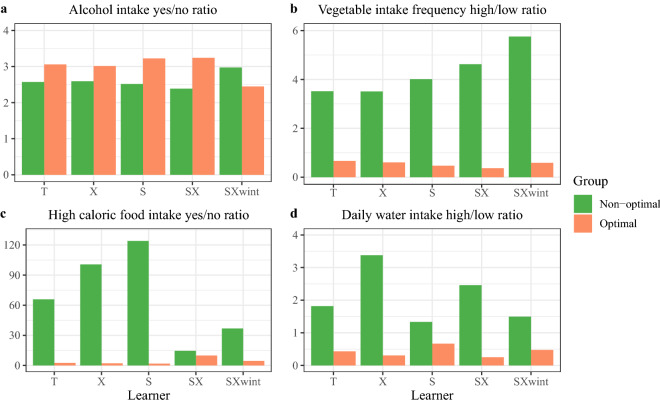
Comparison of sample size ratios between personalized optimal and non-optimal groups for T, X, S, SX and SXwint learners; (**a**) Alcohol *CALC Yes/No Ratio* = (Sample size with CALC = Yes)/(Sample size with CALC = No), (**b**) Vegetable *FCVC High/Low Ratio* = (Sample size with FCVC >2)/(Sample size with FCVC$\le$2), (**c**) High caloric food *FAVC Yes/No Ratio* = (Sample size with FAVC=Yes)/(Sample size with FAVC = No), (**d**) Water CH_2_O High/Low Ratio = (Sample size with CH_2_O > 2)/(Sample size with CH_2_O $\le$ 2). *Optimal* = personalized optimal group. *Non-optimal* = non-optimal group. On the testing data, (**a**) *CALC Yes/No Ratio* = 2.729, (**b**) *FCVC High/Low Ratio* = 1.807, (**c**) *FAVC Yes/No Ratio* = 12.310, (**d**) CH_2_O High/Low Ratio = 0.974.

Individualized optimal nutritional regimens estimated by T, X, S, SX and SXwint learners are all effective in reducing BMI in overweight and obese people. SXwint learner is more effective than SX learner. Depending on the datasets analyzed and the research objectives, the comparison results between these methods are also different. In self obesity management, we find the best-performing metaalgorithms to predict the personalized optimal nutrition lifestyle. In Table 2, SXwint exhibits the largest distance between the distributions of BMI in personalized optimal and non-optimal groups for alcohol, vegetable and daily water intake, as shown by *KS Test 1 D*. On the other hand, T and X learners show the greatest distance between the distributions of BMI in personalized optimal and general optimal groups for alcohol, vegetable and high caloric food intake, as shown by *KS Test 2 D*. Compared to common decisions, personalized optimal decisions estimated by T and X learners tend to give the lowest BMI. In the study of daily water intake, SXwint learner brings the greatest distance between the distributions of BMI in personalized optimal and general optimal groups, and between the distributions of BMI in personalized optimal and non-optimal groups.

### Personalized optimal nutrition lifestyle

On the testing data, the population with positive* alcohol intake* has a higher BMI level than the population with no alcohol intake, as shown in Fig. 3. To reduce BMI, the general better-on-average recommendation is zero alcohol intake for everyone. The general optimal group is the set of all people with zero alcohol intake on the testing data. The BMI distributions of personalized optimal and non-optimal groups are significantly different, where SXwint learner brings the greatest distance and SX learner yields the smallest, as illustrated in Table 2. The BMI distributions of personalized optimal and general optimal groups are also significantly different, where X learner produces the greatest distance and SX learner outputs the smallest. Figure 4 demonstrates that the personalized optimal and non-optimal groups are of comparable Yes/No ratios, which implies that the sample size of positive alcohol intake is approximately 2–3 times the sample size of zero alcohol intake in both personalized optimal and non-optimal groups.

Figure 3 reveals that people with a low* vegetable intake* frequency have lower BMI levels, so the common decision is low-frequency vegetable intake for everyone. The general optimal group is the set of all people with a low vegetable intake frequency on the testing data. The BMI distributions of personalized optimal and non-optimal groups are significantly different, where SXwint learner yields the greatest distance and S learner produces the smallest, as demonstrated in Table 2. The BMI distributions of personalized optimal and general optimal groups are also significantly different, where X learner outputs the greatest distance and SXwint learner shows the smallest. Figure 4 indicates that the FCVC High/Low ratio is much higher in the non-optimal group than in the personalized optimal group. Compared with the non-optimal group, a higher proportion of people in the personalized optimal group consume vegetable at a low frequency.

Figure 1 and Table 1 show that catboost and random forest do not consider FAVC (*high caloric food intake*) important, but covariance test identifies FAVC as significant. Intuitively, the effect of FAVC should be significant, since FAVC = Yes means higher energy intake, and FAVC = No means lower energy intake. The BMI distributions of personalized optimal and non-optimal groups are significantly different, where X learner outputs the greatest distance and SX learner produces the smallest, as shown in Table 2. The general optimal group is the set of all people with a low high-calorie food intake frequency on the testing data. Almost all people in the general optimal group have BMI below 30. Despite that the general advice is already very effective, the personalized optimal solutions estimated by metaalgorithms further reduce the BMI. Table 2 illustrates that the BMI distributions of personalized optimal and general optimal groups are significantly different for all learners except SX learner. Figure 4 shows that the FAVC Yes/No ratio is much higher in the non-optimal group than in the personalized optimal group especially for T, X and S learners. In the personalized optimal group, a much smaller proportion of individuals consume high-calorie foods frequently.

On the testing data, individuals with low* daily water intake* show lower BMI on average, which implies that the general strategy is to drink less than 2 liters of water everyday. The BMI distributions of personalized optimal and non-optimal groups are significantly different, where SXwint learner yields the greatest distance and SX learner produces the smallest, as in Table 2. The BMI distributions of personalized optimal and general optimal groups are also significantly different, where SXwint learner outputs the greatest distance and SX learner returns the smallest. Figure 4 shows that the CH_2_O (daily water intake) High/Low ratio is much higher in the non-optimal group than in the personalized optimal group especially for X and SX learners. A smaller proportion of individuals consume more than 2 liters of water everyday in the personalized optimal group. Figure 1 and Table 1 show that catboost and random forest consider CH_2_O important, but covariance test identifies CH_2_O as insignificant. Intuitively daily water intake has no effect on BMI since it does not affect energy intake or consumption processes. However, in our empirical analysis, individualized optimal nutrition regimens on daily water intake still reduce BMI, as shown in Fig. 3. In carefully designed clinical trials, water intake should have no effect on BMI. But in self obesity management, making daily water intake equal to the personalized optimal decision is beneficial for lowering BMI.

## Conclusion

For overweight and obese people, in order to reduce BMI, the general recommendation is to lower the intake of all foods and beverages: alcohol, vegetables, high caloric foods and water. However, individualized optimal nutritional regimens estimated by metaalgorithms are more effective in reducing BMI. In a personalized optimal regimen, for some populations,  surprisingly, consuming more on a particular type of food or drink is beneficial for lowering BMI. Through calculations, we find that SXwint learner tends to make BMI distributions in personalized optimal and non-optimal groups more distant. On the contrary, T and X learners tend to make BMI distributions in personalized optimal and general optimal groups more distant.

## Data Availability

The data sets analyzed during the current study are available from the corresponding author on reasonable request.

## References

[1] Johnson KB (2021). Precision medicine, AI, and the future of personalized health care. Clin. Trans. Sci..

[2] Gunter L, Zhu J, Murphy S (2011). Variable selection for qualitative interactions in personalized medicine while controlling the family-wise error rate. J. Biopharm. Stat..

[3] Kapelner A (2021). Evaluating the effectiveness of personalized medicine with software. Front. Big Data.

[4] Schulte PJ, Tsiatis AA, Laber EB, Davidian M (2014). $$\mathbf{Q}$$- and $$\mathbf{A}$$-learning methods for estimating optimal dynamic treatment regimes. Stat. Sci..

[5] Zhang B, Tsiatis AA, Laber EB, Davidian M (2012). A robust method for estimating optimal treatment regimes. Biometrics.

[6] Wei D (2021). Dataflow management in the Internet of Things: Sensing, control, and security. Tsinghua Sci. Technol..

[7] Gubbi J, Buyya R, Marusic S, Palaniswami M (2013). Internet of Things (IoT): A vision, architectural elements, and future directions. Fut. Gener. Comput. Syst..

[8] Xu X, Gu R, Dai F, Qi L, Wan S (2020). Multi-objective computation offloading for internet of vehicles in cloud-edge computing. Wirel. Netw..

[9] Li J (2021). Sampling-based approximate skyline query in sensor equipped IoT networks. Tsinghua Sci. Technol..

[10] Wu X (2020). Locally private frequency estimation of physical symptoms for infectious disease analysis in Internet of medical Things. Comput. Commun..

[11] Khan MA, Salah K (2018). IoT security: Review, blockchain solutions, and open challenges. Fut. Gener. Comput. Syst..

[12] Xu X (2020). BeCome: Blockchain-enabled computation offloading for IoT in mobile edge computing. IEEE Trans. Ind. Inf..

[13] Kong L (2021). LSH-aware multitype health data prediction with privacy preservation in edge environment. World Wide Web.

[14] Ren J, Li J, Liu H, Qin T (2022). Task offloading strategy with emergency handling and blockchain security in SDN-empowered and fog-assisted healthcare IoT. Tsinghua Sci. Technol..

[15] Wang X, Zhou Y, Zhao C (2022). Heart-rate analysis of healthy and insomnia groups with detrended fractal dimension feature in edge. Tsinghua Sci. Technol..

[16] San-Cristobal R, Navas-Carretero S, Martínez-González MÁ, Ordovas JM, Martínez JA (2020). Contribution of macronutrients to obesity: Implications for precision nutrition. Nat. Rev. Endocrinol..

[17] Hu G, Tuomilehto J (2007). Lifestyle and outcome among patients with type 2 diabetes. Int. Congr. Ser..

[18] Guglielmo D (2018). Health care provider counseling for weight loss among adults with arthritis and overweight or obesity: United States, 2002–2014. MMWR. Morb. Mortal. Wkly Rep..

[19] Berceanu M, Cheng CW, Viswambharan H, Kain K (2022). Disparity in association of obesity measures with ankle and brachial systolic blood pressures in Europeans and South Asians. Sci. Rep..

[20] Qingxian C (2020). Obesity and COVID-19 severity in a designated hospital in Shenzhen. China. Diabetes Care.

[21] Agarwal A, Sharma S, Kumar V, Kaur M (2021). Effect of E-learning on public health and environment during COVID-19 lockdown. Big Data Min. Anal..

[22] Rodrigues FM, Abreu AM, Holmström I, Mineiro A (2022). E-learning is a burden for the deaf and hard of hearing. Sci. Rep..

[23] Shin JH, Park S, Cho H, Kim JH, Choi H (2022). Adipokine human Resistin promotes obesity-associated inflammatory intervertebral disc degeneration via pro-inflammatory cytokine cascade activation. Sci. Rep..

[24] Westgate CSJ (2022). The impact of obesity-related raised intracranial pressure in rodents. Sci. Rep..

[25] Wu Y, Perng W, Peterson KE (2020). Precision nutrition and childhood obesity: A scoping review. Metabolites.

[26] Hsu P-C, Hwang F-M, Chien M-I, Mui W-C, Lai J-M (2022). The impact of maternal influences on childhood obesity. Sci. Rep..

[27] Abaj F (2022). Interactions between Caveolin-1 polymorphism and Plant-based dietary index on metabolic and inflammatory markers among women with obesity. Sci. Rep..

[28] Malsagova KA (2021). Sports nutrition: Diets, selection factors, recommendations. Nutrients.

[29] Zeevi D (2015). Personalized nutrition by prediction of glycemic responses. Cell.

[30] de Hoogh IM (2021). A novel personalized systems nutrition program improves dietary patterns, lifestyle behaviors and health-related outcomes: Results from the habit study. Nutrients.

[31] Palechor FM, de la Hoz Manotas A (2019). Dataset for estimation of obesity levels based on eating habits and physical condition in individuals from Colombia Peru and Mexico. Data Brief.

[32] Künzel SR, Sekhon JS, Bickel PJ, Yu B (2019). Metalearners for estimating heterogeneous treatment effects using machine learning. Proc. Nat. Acad. Sci..

[33] De La Hoz-Correa E, Mendoza-Palechor FE, De la Hoz-Manotas A, Morales-Ortega RC, Adriana SHB (2019). Obesity level estimation software based on decision trees. J. Comput. Sci..

[34] Dorogush, A. V., Ershov, V. & Gulin, A. CatBoost: Gradient boosting with categorical features support. arXiv:1810.11363 (2018).

[35] Wager S, Athey S (2018). Estimation and inference of heterogeneous treatment effects using random forests. J. Am. Stat. Assoc..

[36] Iwendi C (2020). COVID-19 patient health prediction using boosted random forest algorithm. Front. Publ. Health.

[37] Lockhart R, Taylor J, Tibshirani RJ, Tibshirani R (2014). A significance test for the lasso. Ann. Stat..

[38] Rubin DB (1974). Estimating causal effects of treatments in randomized and non-randomized studies. J. Educ. Psychol..

[39] Rosenbaum PR, Rubin DB (1983). The central role of the propensity score in observational studies for causal effects. Biometrika.

